# 3-(4-Methyl­phen­yl)-4*H*-chromen-4-one

**DOI:** 10.1107/S2414314621005903

**Published:** 2021-06-15

**Authors:** Miri Yoo, Dongsoo Koh

**Affiliations:** a Department of Applied Chemistry, Dongduk Women’s University, Seoul 136-714, Republic of Korea; University of Aberdeen, Scotland

**Keywords:** crystal structure, chromene, isoflavones

## Abstract

In the title compound, C_16_H_12_O_2_, the dihedral angle formed between the plane of the chromenone ring system (r.m.s. deviation = 0.031 Å) and the pendant benzene ring is 31.09 (5)°. In the crystal, weak C—H⋯O hydrogen bonds link the mol­ecules into C(6) chains propagating along the *a*-axis direction.

## Abstract 2

In the title compound, the dihedral angle formed between the plane of the chromenone ring system (r.m.s. deviation = 0.031 Å) and the pendant benzene ring is 31.09 (5)°. In the crystal, weak C—H⋯O hydrogen bonds link the mol­ecules into C(6) chains propagating along the *a*-axis direction.

## Graphical abstract

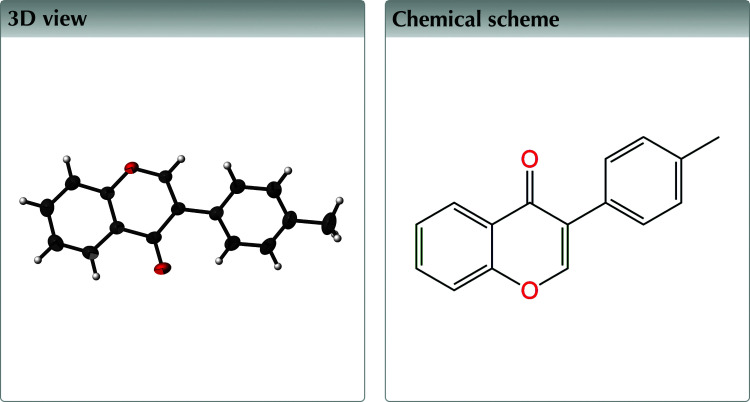

## Structure description

Isoflavones are flavonoid polyphenols with a general C6—C3—C6 carbon-atom skeleton. Since isoflavones have a substituent at the 2-position of the flavonoid skeleton, they exhibit structural differences from other compounds belonging to flavonoids having a substituent at the 3-position. Isoflavones are sometimes classified as phytoestrogens, and they also exhibit different physiological functions from other flavonoids (Tikkanen *et al.*, 2000 ▸). Recent research has shown that isoflavones have broad biological activities with resspect to osteoporosis (Ye *et al.*, 2006 ▸), anti­cancer activity (Messina *et al.*, 2009 ▸), cardiovascular diseases (Zhan *et al.*, 2005 ▸) and the inhibition of thyroid peroxidase (Chang *et al.*, 2000 ▸). As part of our ongoing studies of isoflavones (Ahn *et al.*, 2020 ▸; Shin *et al.*, 2020 ▸), the title compound was synthesized and its crystal structure was determined.

**Figure 1:**
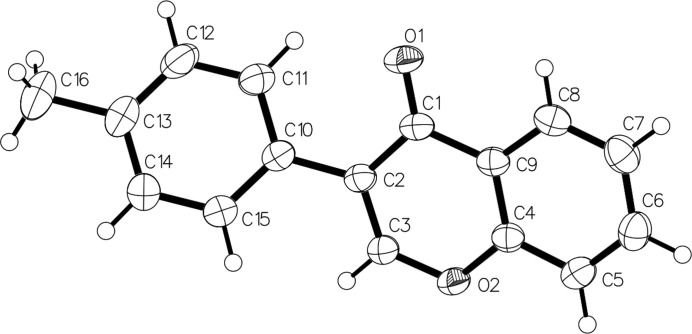
The mol­ecular structure of the title compound with displacement ellipsoids drawn at the 30% probability level.

**Figure 2:**
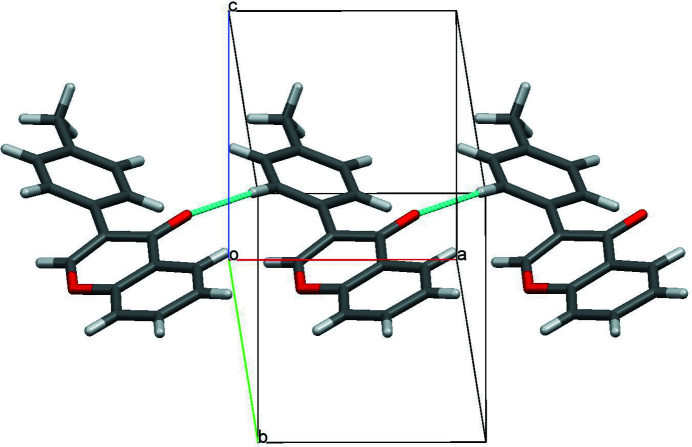
Part of the crystal structure of the title compound with C—H⋯O hydrogen bonds shown as dashed lines.

The mol­ecular structure of the title compound, C_16_H_12_O_2_, is shown in Fig. 1 ▸. The chromenone ring system (C1–C9/O2) is slightly twisted from planarity, with a maximum deviation of 0.059 Å at C2 (root–mean–square deviation = 0.031 Å). The dihedral angle formed between the mean plane of the chromenone ring system and the pendant benzene (C10–C15) ring is 31.09 (5)°. In the crystal, weak C—H⋯O hydrogen bonds link the mol­ecules into *C*(6) chains propagating along the *a*-axis direction (Table 1 ▸, Fig. 2 ▸).

## Synthesis and crystallization

**Figure 3:**
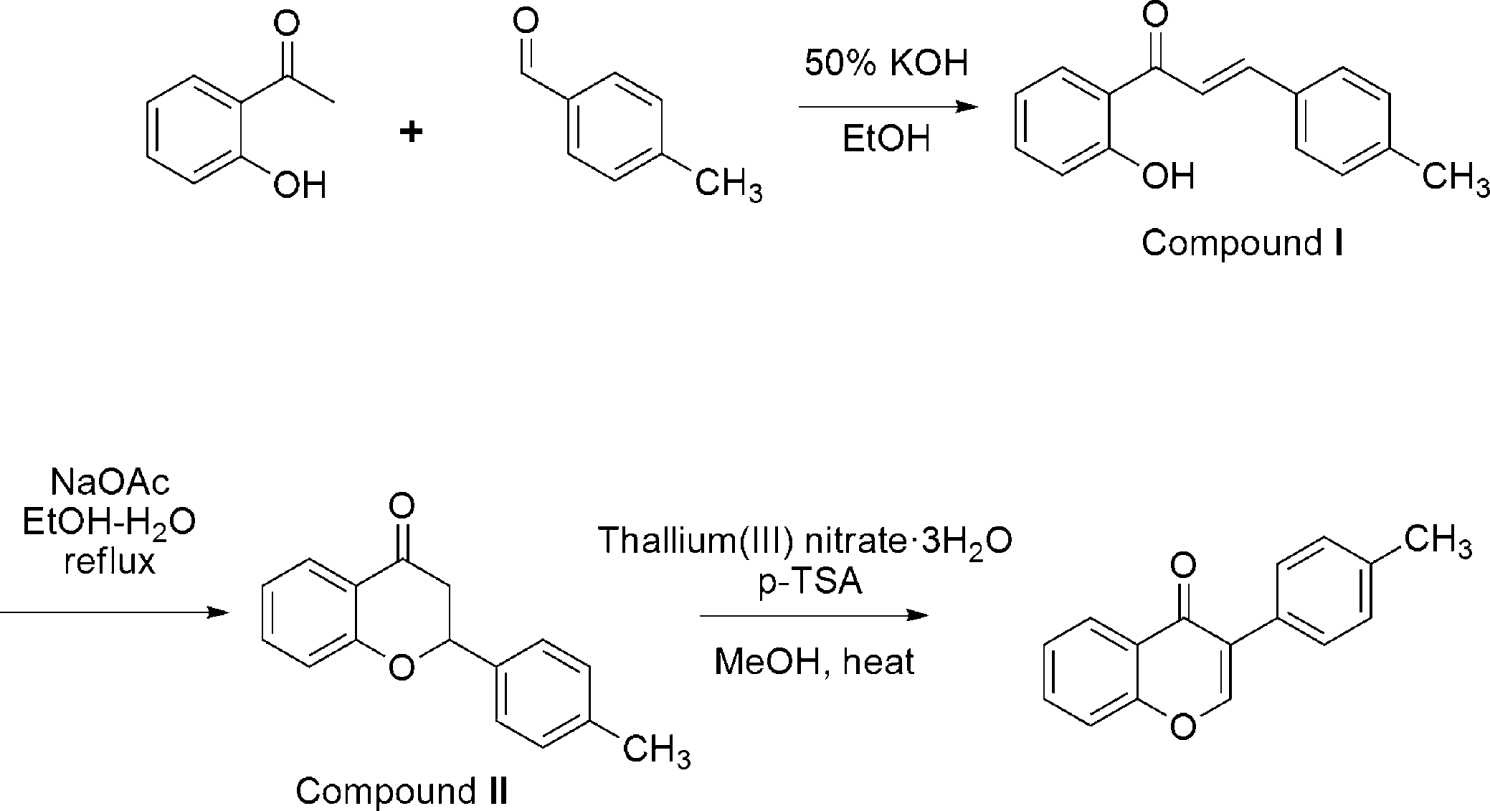
Synthetic scheme for the preparation of the title compound.

The title compound was synthesized in three steps from the commercially available starting materials 2-hydoxyaceto­phenone and 4-methyl­benzaldehyde according to the reaction scheme shown in Fig. 3 ▸. To a solution of 2-hydoxy­aceto­phenone (408 mg, 3 mmol) in 40 ml of ethanol was added 4-methyl­benzaldehyde (360 mg, 3 mmol) and the temperature was adjusted to around 277 K in an ice-bath. To the cooled reaction mixture were added 4 ml of 30% aqueous KOH solution and the reaction mixture was stirred at room temperature for 3 h. This mixture was poured into iced water (100 ml) and was acidified (pH = 2) with 2 *M* HCl solution to give a precipitate. Filtration and washing with water afforded crude solid of chalcone compound (**I**). To a solution of **I** (1.5 mmol, 357 mg) in 20 ml aqueous ethanol (H_2_O:ethanol = 1:2) was added excess sodium acetate and the solution was refluxed at 362 K for 2 h. The reaction mixture was cooled to room temperature and was poured into iced water (50 ml) to give a precipitate of the flavanone compound **II**. Compound **II** (163 mg, 0.5 mmol) was dissolved in 15 ml of methanol and the temperature was adjusted to around 327 K. To the clear solution were added catalytic amount of *p*-toluene sulfonic acid and 1.2eq of thallium(III) nitrate trihydrate and the mixture was refluxed for 5 h. The reaction mixture was cooled to room temperature and the resulting precipitate was filtered and washed with water. This solid was recrystallized from an ethanol solution to obtain single crystals of the title compound.

## Refinement

Crystal data, data collection and structure refinement details are summarized in Table 2 ▸.

## Supplementary Material

Crystal structure: contains datablock(s) I. DOI: 10.1107/S2414314621005903/hb4386sup1.cif

Structure factors: contains datablock(s) I. DOI: 10.1107/S2414314621005903/hb4386Isup2.hkl

Supporting information file. DOI: 10.1107/S2414314621005903/hb4386Isup3.cml

CCDC reference: 2088523

Additional supporting information:  crystallographic information; 3D view; checkCIF report

## Figures and Tables

**Table 1:** Hydrogen-bond geometry (Å, °)

*D*—H⋯*A*	*D*—H	H⋯*A*	*D*⋯*A*	*D*—H⋯*A*
C15—H15⋯O1^i^	0.94	2.49	3.4255 (16)	172

Symmetry code: (i) 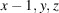 .

**Table 2:** Experimental details

Crystal data	
Chemical formula	C_16_H_12_O_2_
*M*_r_	236.26
Crystal system, space group	Triclinic, *P* 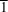
Temperature (K)	223
*a*, *b*, *c* (Å)	6.4514 (3), 7.0785 (4), 13.3144 (7)
α, β, γ (°)	78.906 (2), 85.276 (2), 79.628 (2)
*V* (Å^3^)	586.19 (5)
*Z*	2
Radiation type	Mo *K*α
μ (mm^−1^)	0.09
Crystal size (mm)	0.56 × 0.31 × 0.12

Data collection	
Diffractometer	Bruker PHOTON II M14 CCD
Absorption correction	Multi-scan (*SADABS*; Bruker, 2012 ▸)
*T*_min_, *T*_max_	0.666, 0.746
No. of measured, independent and observed [*I* > 2σ(*I*)] reflections	18172, 2904, 2459
*R*_int_	0.032
(sin θ/λ)_max_ (Å^−1^)	0.668

Refinement	
*R*[*F* ^2^ > 2σ(*F* ^2^)], *wR*(*F* ^2^), *S*	0.044, 0.124, 1.06
No. of reflections	2904
No. of parameters	164
H-atom treatment	H-atom parameters constrained
Δρ_max_, Δρ_min_ (e Å^−3^)	0.27, −0.22

Computer programs: *APEX2* and *SAINT* (Bruker, 2012 ▸), *SHELXS* (Sheldrick, 2008 ▸), *SHELXL2014/7* (Sheldrick, 2015 ▸), *SHELXTL* (Sheldrick, 2008 ▸) and *publCIF* (Westrip, 2010 ▸).

## full crystallographic data

### Crystal data

**Table:**

C_16_H_12_O_2_	*Z* = 2
*M**_r_* = 236.26	*F*(000) = 248
Triclinic, *P*1	*D*_x_ = 1.339 Mg m^−^^3^
*a* = 6.4514 (3) Å	Mo *K*α radiation, λ = 0.71073 Å
*b* = 7.0785 (4) Å	Cell parameters from 7663 reflections
*c* = 13.3144 (7) Å	θ = 3.0–28.3°
α = 78.906 (2)°	µ = 0.09 mm^−^^1^
β = 85.276 (2)°	*T* = 223 K
γ = 79.628 (2)°	Block, colourless
*V* = 586.19 (5) Å^3^	0.56 × 0.31 × 0.12 mm

### Data collection

**Table:**

Bruker PHOTON II M14 CCD diffractometer	2459 reflections with *I* > 2σ(*I*)
φ and ω scans	*R*_int_ = 0.032
Absorption correction: multi-scan (SADABS; Bruker, 2012)	θ_max_ = 28.3°, θ_min_ = 3.0°
*T*_min_ = 0.666, *T*_max_ = 0.746	*h* = −8→8
18172 measured reflections	*k* = −9→9
2904 independent reflections	*l* = −17→17

### Refinement

**Table:**

Refinement on *F*^2^	Primary atom site location: structure-invariant direct methods
Least-squares matrix: full	Hydrogen site location: inferred from neighbouring sites
*R*[*F*^2^ > 2σ(*F*^2^)] = 0.044	H-atom parameters constrained
*wR*(*F*^2^) = 0.124	*w* = 1/[σ^2^(*F*_o_^2^) + (0.0506*P*)^2^ + 0.2032*P*] where *P* = (*F*_o_^2^ + 2*F*_c_^2^)/3
*S* = 1.06	(Δ/σ)_max_ < 0.001
2904 reflections	Δρ_max_ = 0.27 e Å^−^^3^
164 parameters	Δρ_min_ = −0.22 e Å^−^^3^
0 restraints	

### Special details

**Table:**

Geometry. All esds (except the esd in the dihedral angle between two l.s. planes) are estimated using the full covariance matrix. The cell esds are taken into account individually in the estimation of esds in distances, angles and torsion angles; correlations between esds in cell parameters are only used when they are defined by crystal symmetry. An approximate (isotropic) treatment of cell esds is used for estimating esds involving l.s. planes.

### Fractional atomic coordinates and isotropic or equivalent isotropic displacement parameters (Å^2^)

**Table:**

	*x*	*y*	*z*	*U*_iso_*/*U*_eq_	
C1	0.56922 (19)	0.72771 (17)	0.62667 (10)	0.0304 (3)	
O1	0.72856 (15)	0.67806 (17)	0.67655 (8)	0.0452 (3)	
C2	0.35627 (19)	0.78209 (17)	0.67070 (9)	0.0290 (3)	
C3	0.19573 (19)	0.84130 (19)	0.60689 (9)	0.0318 (3)	
H3	0.0619	0.8826	0.6365	0.038*	
O2	0.20859 (13)	0.84701 (14)	0.50433 (7)	0.0338 (2)	
C4	0.40159 (19)	0.78707 (17)	0.45916 (9)	0.0294 (3)	
C5	0.4074 (2)	0.7839 (2)	0.35475 (10)	0.0365 (3)	
H5	0.2836	0.8215	0.3182	0.044*	
C6	0.5979 (2)	0.7246 (2)	0.30617 (11)	0.0408 (3)	
H6	0.6040	0.7197	0.2359	0.049*	
C7	0.7824 (2)	0.6717 (2)	0.36019 (11)	0.0416 (3)	
H7	0.9121	0.6326	0.3263	0.050*	
C8	0.7736 (2)	0.67708 (19)	0.46305 (11)	0.0361 (3)	
H8	0.8984	0.6430	0.4989	0.043*	
C9	0.58123 (19)	0.73260 (16)	0.51535 (9)	0.0289 (3)	
C10	0.3097 (2)	0.77022 (18)	0.78255 (9)	0.0319 (3)	
C11	0.4540 (2)	0.8019 (2)	0.84770 (11)	0.0408 (3)	
H11	0.5867	0.8302	0.8209	0.049*	
C12	0.4033 (3)	0.7920 (2)	0.95158 (11)	0.0467 (4)	
H12	0.5035	0.8125	0.9940	0.056*	
C13	0.2093 (3)	0.7528 (2)	0.99470 (11)	0.0450 (3)	
C14	0.0661 (2)	0.7211 (2)	0.93000 (11)	0.0469 (4)	
H14	−0.0672	0.6952	0.9570	0.056*	
C15	0.1159 (2)	0.7269 (2)	0.82625 (10)	0.0400 (3)	
H15	0.0173	0.7012	0.7846	0.048*	
C16	0.1555 (3)	0.7426 (3)	1.10810 (12)	0.0646 (5)	
H16A	0.2235	0.6185	1.1454	0.097*	
H16B	0.0038	0.7546	1.1206	0.097*	
H16C	0.2046	0.8482	1.1310	0.097*	

### Atomic displacement parameters (Å^2^)

**Table:**

	*U*^11^	*U*^22^	*U*^33^	*U*^12^	*U*^13^	*U*^23^
C1	0.0281 (6)	0.0288 (6)	0.0353 (6)	−0.0060 (4)	−0.0082 (5)	−0.0047 (5)
O1	0.0290 (5)	0.0644 (7)	0.0421 (5)	−0.0023 (4)	−0.0131 (4)	−0.0094 (5)
C2	0.0293 (6)	0.0282 (6)	0.0307 (6)	−0.0069 (4)	−0.0057 (4)	−0.0047 (4)
C3	0.0274 (6)	0.0379 (6)	0.0298 (6)	−0.0038 (5)	−0.0036 (4)	−0.0062 (5)
O2	0.0263 (4)	0.0444 (5)	0.0301 (4)	−0.0018 (4)	−0.0074 (3)	−0.0066 (4)
C4	0.0288 (6)	0.0273 (6)	0.0328 (6)	−0.0054 (4)	−0.0038 (5)	−0.0058 (4)
C5	0.0398 (7)	0.0375 (7)	0.0338 (6)	−0.0075 (5)	−0.0081 (5)	−0.0068 (5)
C6	0.0504 (8)	0.0404 (7)	0.0335 (6)	−0.0100 (6)	0.0016 (6)	−0.0106 (5)
C7	0.0389 (7)	0.0401 (7)	0.0460 (8)	−0.0056 (6)	0.0061 (6)	−0.0133 (6)
C8	0.0296 (6)	0.0344 (6)	0.0446 (7)	−0.0040 (5)	−0.0030 (5)	−0.0085 (5)
C9	0.0290 (6)	0.0249 (5)	0.0337 (6)	−0.0060 (4)	−0.0046 (5)	−0.0048 (4)
C10	0.0344 (6)	0.0315 (6)	0.0304 (6)	−0.0056 (5)	−0.0066 (5)	−0.0047 (5)
C11	0.0408 (7)	0.0481 (8)	0.0369 (7)	−0.0140 (6)	−0.0080 (6)	−0.0078 (6)
C12	0.0553 (9)	0.0543 (9)	0.0347 (7)	−0.0139 (7)	−0.0145 (6)	−0.0088 (6)
C13	0.0572 (9)	0.0460 (8)	0.0302 (7)	−0.0050 (7)	−0.0050 (6)	−0.0054 (6)
C14	0.0421 (8)	0.0615 (10)	0.0356 (7)	−0.0105 (7)	0.0009 (6)	−0.0048 (6)
C15	0.0356 (7)	0.0516 (8)	0.0341 (7)	−0.0112 (6)	−0.0052 (5)	−0.0058 (6)
C16	0.0831 (13)	0.0786 (13)	0.0306 (8)	−0.0110 (10)	−0.0025 (8)	−0.0086 (8)

### Geometric parameters (Å, º)

**Table:**

C1—O1	1.2304 (15)	C8—C9	1.4026 (18)
C1—C2	1.4638 (17)	C8—H8	0.9400
C1—C9	1.4719 (17)	C10—C15	1.3947 (18)
C2—C3	1.3497 (16)	C10—C11	1.3974 (18)
C2—C10	1.4840 (17)	C11—C12	1.386 (2)
C3—O2	1.3544 (15)	C11—H11	0.9400
C3—H3	0.9400	C12—C13	1.385 (2)
O2—C4	1.3699 (15)	C12—H12	0.9400
C4—C9	1.3865 (16)	C13—C14	1.388 (2)
C4—C5	1.3919 (17)	C13—C16	1.512 (2)
C5—C6	1.376 (2)	C14—C15	1.3866 (19)
C5—H5	0.9400	C14—H14	0.9400
C6—C7	1.397 (2)	C15—H15	0.9400
C6—H6	0.9400	C16—H16A	0.9700
C7—C8	1.374 (2)	C16—H16B	0.9700
C7—H7	0.9400	C16—H16C	0.9700
			
O1—C1—C2	124.34 (12)	C4—C9—C1	121.04 (11)
O1—C1—C9	120.97 (12)	C8—C9—C1	121.16 (11)
C2—C1—C9	114.68 (10)	C15—C10—C11	117.53 (12)
C3—C2—C1	118.45 (11)	C15—C10—C2	120.11 (11)
C3—C2—C10	118.92 (11)	C11—C10—C2	122.36 (12)
C1—C2—C10	122.62 (10)	C12—C11—C10	120.57 (13)
C2—C3—O2	126.36 (11)	C12—C11—H11	119.7
C2—C3—H3	116.8	C10—C11—H11	119.7
O2—C3—H3	116.8	C13—C12—C11	121.94 (13)
C3—O2—C4	118.06 (9)	C13—C12—H12	119.0
O2—C4—C9	121.22 (11)	C11—C12—H12	119.0
O2—C4—C5	116.67 (11)	C12—C13—C14	117.48 (13)
C9—C4—C5	122.11 (12)	C12—C13—C16	121.51 (15)
C6—C5—C4	118.67 (12)	C14—C13—C16	121.00 (15)
C6—C5—H5	120.7	C15—C14—C13	121.28 (14)
C4—C5—H5	120.7	C15—C14—H14	119.4
C5—C6—C7	120.67 (13)	C13—C14—H14	119.4
C5—C6—H6	119.7	C14—C15—C10	121.18 (13)
C7—C6—H6	119.7	C14—C15—H15	119.4
C8—C7—C6	119.77 (13)	C10—C15—H15	119.4
C8—C7—H7	120.1	C13—C16—H16A	109.5
C6—C7—H7	120.1	C13—C16—H16B	109.5
C7—C8—C9	120.98 (12)	H16A—C16—H16B	109.5
C7—C8—H8	119.5	C13—C16—H16C	109.5
C9—C8—H8	119.5	H16A—C16—H16C	109.5
C4—C9—C8	117.77 (12)	H16B—C16—H16C	109.5
			
O1—C1—C2—C3	177.41 (13)	C7—C8—C9—C1	176.44 (12)
C9—C1—C2—C3	−3.73 (16)	O1—C1—C9—C4	179.50 (12)
O1—C1—C2—C10	−4.05 (19)	C2—C1—C9—C4	0.59 (16)
C9—C1—C2—C10	174.81 (10)	O1—C1—C9—C8	1.32 (18)
C1—C2—C3—O2	3.58 (19)	C2—C1—C9—C8	−177.58 (11)
C10—C2—C3—O2	−175.02 (11)	C3—C2—C10—C15	30.64 (18)
C2—C3—O2—C4	0.20 (19)	C1—C2—C10—C15	−147.90 (13)
C3—O2—C4—C9	−3.63 (17)	C3—C2—C10—C11	−149.10 (13)
C3—O2—C4—C5	176.38 (11)	C1—C2—C10—C11	32.37 (19)
O2—C4—C5—C6	−179.99 (12)	C15—C10—C11—C12	−0.6 (2)
C9—C4—C5—C6	0.01 (19)	C2—C10—C11—C12	179.13 (13)
C4—C5—C6—C7	−1.0 (2)	C10—C11—C12—C13	−0.7 (2)
C5—C6—C7—C8	0.6 (2)	C11—C12—C13—C14	0.7 (2)
C6—C7—C8—C9	0.8 (2)	C11—C12—C13—C16	−179.92 (16)
O2—C4—C9—C8	−178.63 (11)	C12—C13—C14—C15	0.5 (2)
C5—C4—C9—C8	1.36 (18)	C16—C13—C14—C15	−178.87 (15)
O2—C4—C9—C1	3.14 (18)	C13—C14—C15—C10	−1.8 (2)
C5—C4—C9—C1	−176.87 (11)	C11—C10—C15—C14	1.8 (2)
C7—C8—C9—C4	−1.79 (19)	C2—C10—C15—C14	−177.94 (13)

### Hydrogen-bond geometry (Å, º)

**Table:**

*D*—H···*A*	*D*—H	H···*A*	*D*···*A*	*D*—H···*A*
C15—H15···O1^i^	0.94	2.49	3.4255 (16)	172

Symmetry code: (i) *x*−1, *y*, *z*.
